# RMND5 from *Xenopus laevis* Is an E3 Ubiquitin-Ligase and Functions in Early Embryonic Forebrain Development

**DOI:** 10.1371/journal.pone.0120342

**Published:** 2015-03-20

**Authors:** Thorsten Pfirrmann, Pablo Villavicencio-Lorini, Abinash K. Subudhi, Ruth Menssen, Dieter H. Wolf, Thomas Hollemann

**Affiliations:** 1 Martin-Luther University Halle-Wittenberg, Institute of Physiological Chemistry, Halle, Germany; 2 Martin Luther University Halle-Wittenberg, Institute of Human Genetics, Halle, Germany; 3 University of Stuttgart, Institute of Biochemistry, Pfaffenwaldring 55, 70569 Stuttgart, Germany; School of Biomedical Sciences, The University of Queensland, AUSTRALIA

## Abstract

In *Saccharomyces cerevisiae* the Gid-complex functions as an ubiquitin-ligase complex that regulates the metabolic switch between glycolysis and gluconeogenesis. In higher organisms six conserved Gid proteins form the CTLH protein-complex with unknown function. Here we show that Rmnd5, the Gid2 orthologue from Xenopus laevis, is an ubiquitin-ligase embedded in a high molecular weight complex. Expression of *rmnd5* is strongest in neuronal ectoderm, prospective brain, eyes and ciliated cells of the skin and its suppression results in malformations of the fore- and midbrain. We therefore suggest that *Xenopus laevis* Rmnd5, as a subunit of the CTLH complex, is a ubiquitin-ligase targeting an unknown factor for polyubiquitination and subsequent proteasomal degradation for proper fore- and midbrain development.

## Introduction

Previously, we isolated nine GID (glucose-induced degradation deficient) genes necessary for the glucose-induced degradation of fructose-1,6-bisphosphatase (FBPase) from yeast [1]. Seven of these proteins are part of a high molecular mass protein complex. In *Saccharomyces cerevisiae* the so-called Gid-complex functions as an ubiquitin-ligase complex that targets key gluconeogenic enzymes for polyubiquitination and subsequent proteasomal degradation to initiate an irreversible shutdown of the gluconeogenic pathway [2–4].

The ubiquitin proteasome system (UPS) regulates the targeted temporal and spatial degradation of proteins. The covalent modification of a protein with ubiquitin requires a set of three classes of enzymes, the ubiquitin-activating enzyme (E1), the ubiquitin-conjugating enzyme (E2) and finally the ubiquitin-ligase (E3) [5]. In humans there exist an estimated number of 600 ubiquitin-ligases that all share the E3 typical RING or HECT domain. Classical RING domains bind two zinc atoms by a Cys3HCys4 motif [6]. The *Saccharomyces cerevisiae* Gid complex contains two proteins with non-canonical RING domains Gid2 and Gid9, the orthologs of RMND5 and MAEA of the vertebrate CTLH complex [3, 4].

Most subunits of the Gid-complex are conserved with a remarkable sequence and domain-distribution conservation throughout the eukaryotic kingdom [7]. GID1, GID2, GID4, GID5, GID7, GID8 and GID9 have their closest human relatives in RanBP9, RMND5, C17ORF39, ARMC8, MKLN1, C20ORF1 (aka Twa1) and MAEA respectively. All subunits except C17ORF39 are part of the human CTLH-complex [8, 9] and a similar complex in *Arabidopsis thaliana* [10] and other eukaryotes [7] exists.

While function and topology of the yeast Gid-complex subunits are well described, the function of the vertebrate counterpart remains poorly understood. Most subunits carry a CTLH-domain (C-terminal to LisH motif domain) and a LisH-domain (lis homology domain) [11]. The latter is often associated with syndromes involving malformations of the central nervous system [12]. For example, the mammalian CTLH/LisH subunit MKLN1, the ortholog of yeast Gid7 [4, 8], is expressed in the hippocampus and the cerebellum [13] and was recently found to be involved in dynein dependent GABA_A_- receptor transport along actin filaments and microtubuli [14]. A similar function is described for ARMC8 [15].

RMND5A the human ortholog of yeast Gid2 also contains a CTLH/LisH- and the E3 typical RING-domain. Interestingly, mammals encode for two *GID2* orthologous genes called *RMND5A* and *RMND5B*, but only RMND5A is part of the CTLH-complex. Nevertheless, RMND5B associates with several ubiquitin-conjugating enzymes [16]. Therefore it is likely that both proteins function as ubiquitin-ligases. A recent report describes human *RMND5A* expression to be regulated by the small non-coding RNA miRNA-138. In conjunction with RanBP10 and Exportin5, RMND5A is described to protect Exportin5 from proteasomal degradation [17]. Clinical data concerning RMND5A are restricted to a recent case report that describes a giant occipitoparietal meningoencephalocele in a newborn baby. This rare neurodevelopmental defect coincides with a 80.65-kb gain within chromosome band 2p11.2 resulting in a duplication of the *RMND5A* gene [18].

We show that *Xenopus laevis* Rmnd5 functions as an ubiquitin ligase, most probably in the context of the CTLH-complex. *Rmnd5* transcript is mainly present in ectodermal derivatives in *Xenopus laevis*. The suppression of *rmnd5* function leads to defects of the prospective prosencephalon and mesencephalon, pointing towards a role of Rmnd5 during neural development. We speculate that Rmnd5 targets a yet unknown neurospecific factor for degradation.

## Results and Discussion

### *Xenopus laevis* Rmnd5 protein is structurally and functionally related to human RMND5A

**Fig 1:**
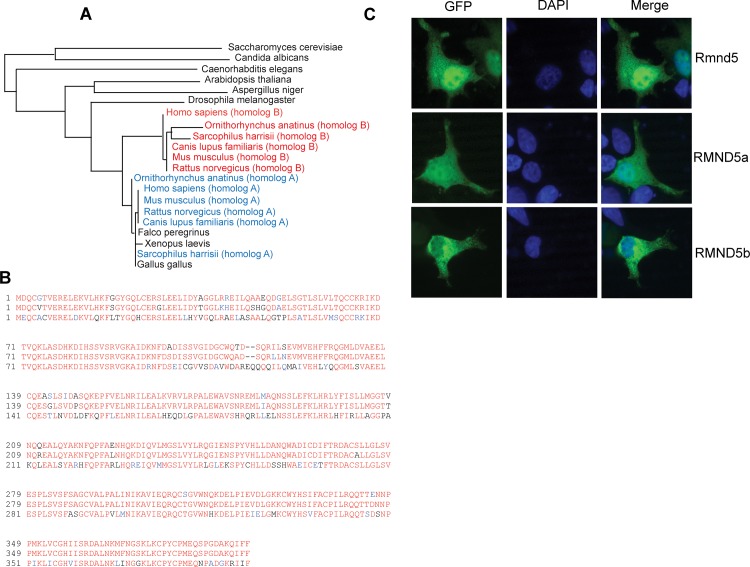
*Xenopus laevis* Rmnd5 protein is structurally and functionally related to human RMND5A. (A) Phylogenetic tree of Rmnd5 orthologs. The taxonomic tree of representative eukaryotic species rendered by Phylogeny.fr software [20]. Respective Gid2/Rmnd5 sequences obtained from NCBI with indicated accession numbers (*Saccharomyces cerevisiae* [NP_010541.3], *Candida albicans* [XP_712238.1], *Aspergillus niger* [XP_001388791.2], *Caenorhabditis elegans* [NP_508444.1], *Arabidopsis thaliana* [NP_196525.1], *Drosophila melanogaster* [NP_611536.3], *Xenopus laevis* [NP_001086276.1], *Falco peregrinus* [XP_005229906.1], *Gallus gallus* [XP_004936301.1] *Homo sapiens* [NP_073617.1; NP_073599.2], *Ornithorhynchus anatinus* [XP_007670084.1; XP_001515875.2], *Sarcophilus harrisii* [XP_003758697.1; XP_003756956.1], *Canis lupus familiaris* [XP_852129.1; XP_531873.2], *Mus musculus* [NP_077250.2; NP_079622.1], *Rattus norvegicus* [XP_232051.4; NP_001017473.1]); homolog A (blue), homolog B (red). (B) Sequence alignment of *Xenopus laevis* Rmnd5 (top), *Homo sapiens* RMND5A (middle) and RMND5B (bottom). Identical residues (red), similar residues (blue), others (black). Identities (%): *Xenopus laevis* Rmnd5 to human RMND5A (94%), to human RMND5B (70%). (C) Localization of *Homo sapiens* RMND5A (RMND5a, middle panel), RMND5B (RMND5b, bottom panel) and *Xenopus laevis* Rmnd5 (Rmnd5, top panel) in HEK293 cells. GFP signal (left column), DAPI signal (middle column), merged signals (right column).

We set out to gain knowledge of the phylogenetic distribution of yeast Gid2 orthologous proteins by comparison of the protein sequences. The Gid2/Rmnd5 protein sequences of the indicated organisms were obtained from NCBI and used for multiple sequence analysis with the T-Coffee algorithm [19]. Further reconstruction and analysis of phylogenetic relationships were done by the Phylogeny.fr software [20] and are displayed in Fig. 1A. It can be seen that Gid2/Rmnd5 is conserved throughout the eukaryotic kingdom. In fungi (*Saccharomyces cerevisiae*, *Candida albicans*, *Aspergillus niger*), nematodes (*Caenorhabditis elegans*), plants (*Arabidopsis thaliana*), insects (*Drosophila melanogaster*), amphibians (*Xenopus laevis*) and birds (*Falco peregrinus*, *Gallus gallus*) only a single isoform is present (shown in black), while mammals (*Homo sapiens*, *Ornithorhynchus anatinus*, *Sarcophilus harrisii*, *Canis lupus*, *Mus musculus*, *Rattus norvegicus* possess two isoforms (homolog A in blue, homolog B in red) that are highly similar. Interestingly, the single isoforms existing in *Xenopus laevis*, *Gallus gallus* and *Falco peregrinus* are phylogenetically closer to the mammalian isoform A (Fig. 1B). While the human RMND5A is 94% identical to *Xenopus laevis* Rmnd5, the human RMND5B isoform only shows 70% identity (calculated by T-Coffee). In previous studies, we have shown that human RMND5A localises to the cytosol and to the nucleus, while human RMND5B is entirely cytosolic [21]. In Fig. 1C representative cells transfected with either *Xenopus laevis rmnd5* (Rmnd5, upper panel), human *RMND5A* (RMND5A, middle panel) or *RMND5B* (RMND5B) can be seen. Respective protein localisation is shown in the left column (GFP), the nuclei in the middle (DAPI) and an overlay in the right column (merge). As shown before, human RMND5A is distributed to the cytosol and the nucleus, while human RMND5B is cytosolic. *Xenopus laevis* Rmnd5 shows a localisation pattern similar to human RMND5A and therefore is not only structurally but also functionally closer to human RMND5A.

Interestingly, two isoforms of RMND5 only exist in mammals with the genomic location of the human *RMND5A* gene on chromosome 2 (2p11.2) and the *RMND5B* gene on chromosome 5 (5q35.3). The appearance of *RMND5B* is therefore a relatively late event in evolution and most probably a consequence of gene duplication. Despite the high degree of structural conservation, it is not clear if both proteins have redundant, overlapping or completely different functions in the cell. It is also unclear if both isoforms can be part of the CTLH-complex as exchangeable subunits. Therefore, to avoid redundancy effects between RMND5A and RMND5B, we consider *Xenopus laevis* as an ideal model organism to study Rmnd5 function during early development.

### *Rmnd5* is expressed during early embryonic development

**Fig 2:**
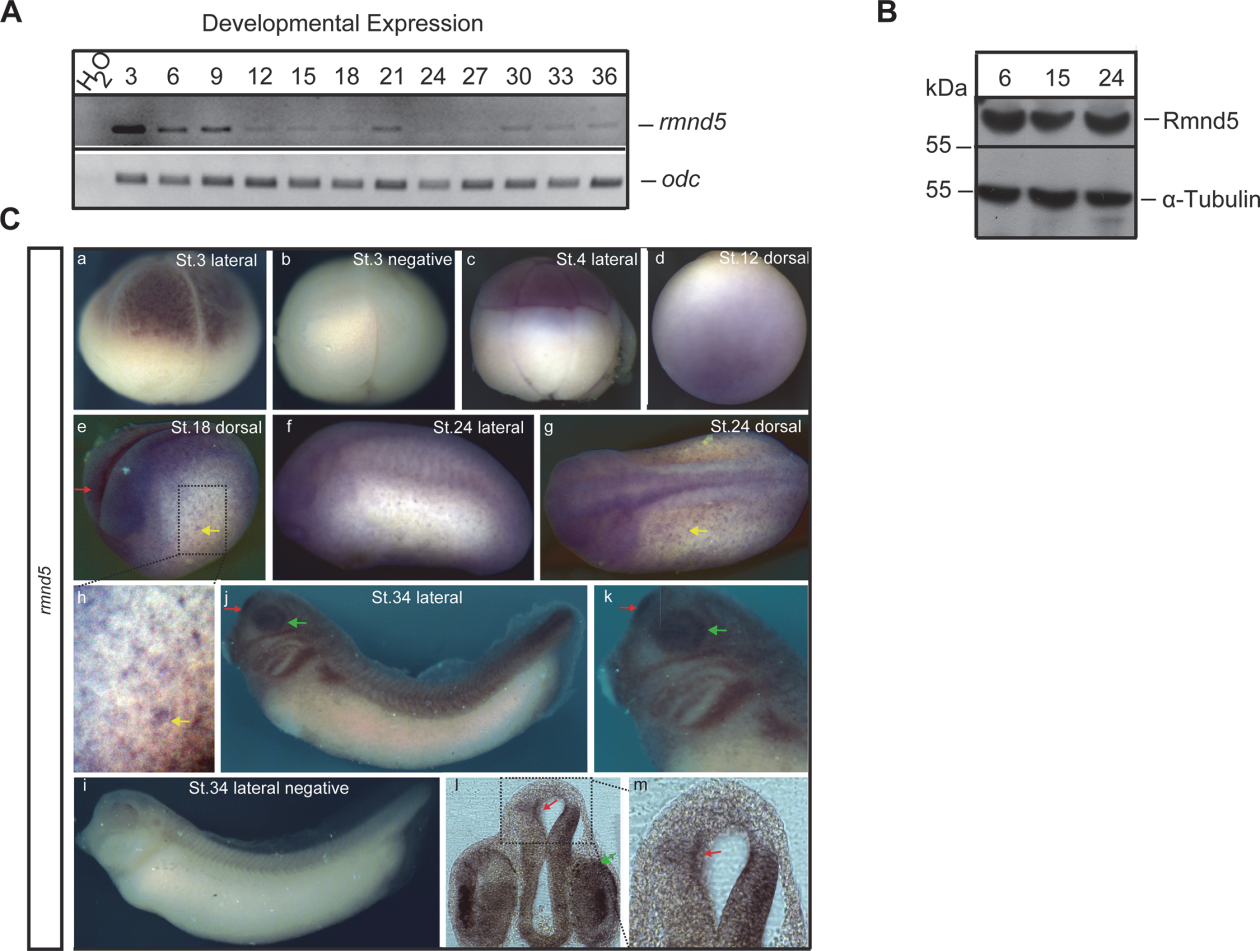
*rmnd5* is expressed during early embryonic development. (A) Temporal RT-PCR analysis of *rmnd5* expression (top panel); different developmental stages (NF-stages) indicated at the top. ODC1 functions as RNA input control (bottom). (B) Rmnd5 protein at different developmental stages. Western blot analysis of embryo lysate from indicated stages (top). α-RMND5A (Novus Biological; rabbit, 1:1000); α-Tubulin (AbD Serotec, rat, 1:2500). (C) Spatial analysis of *rmnd5* expression. Whole mount *in situ* hybridisation (Wmish) of wild type *Xenopus laevis* embryos at different developmental stages. NF stage 3 (panel a, left) and stage 4 (panel c) *rmnd5* transcript in the animal pole (top), NF-stage 12 (panel d) *rmnd5* transcripts around the prospective head, NF-stage 18; 24 (panel e, f, g, h) neuronal ectoderm (red arrow, panel e) and ciliated cells of the skin (yellow arrow, panel e, g, h), NF-stage 34 (panel j, k, l, m) proencephalon (red arrow) and eyes (green arrow). Negative controls with sense probes (panel b, i).

To study a potential role of Rmnd5 during development of the neural system, we first analyzed its spatio-temporal expression pattern in embryos of *Xenopus laevis*. We started out looking at the temporal expression of *rmnd5* by semi-quantitative RT-PCR using total RNA from consecutive developmental stages of *Xenopus laevis* embryos. In Fig. 2A a specific signal at the expected size of 305 bp indicated the existence of *rmnd5* transcript (upper panel), while ODC1 primers were used to control the amount of input RNA (lower panel). The expression of *rmnd5* receives a strong maternal contribution, as RT-PCR products are detectable at NF (Nieuwkoop and Faber) stage 3 and declines steadily until gastrulation (NF stage 12). Zygotic transcripts of *rmnd5* are detectable through all further stages analyzed, though on a much lower level compared to NF stage 3. This finding is confirmed on the protein level, but a comparable decline in Rmnd5 levels is not visible (Fig. 2B). Whole mount *in situ* hybridisation (WMISH) reveals first expression of *rmnd5* at the four-cell stage (Fig. 2C-A, NF stage 3) and the eight-cell stage (Fig. 2C-C, NF stage 4). Here *rmnd5* transcript is mostly present in the animal pole (top), an area developing into derivatives of ectoderm and mesoderm. During gastrulation (NF-stage 12) increasing levels of *rmnd5* transcripts appear enhanced in the prospective head region (Fig. 2C-D) and during neurulation (Fig. 2C-E, NF stage 18) in the neuronal ectoderm (red arrow) and subpopulations of skin cells, most likely the ciliated cells of the skin (Fig. 2C-E, -H; yellow arrow). In a later stage of development (NF stage 34, Fig. 2C-J, -K), the expression of *rmnd5* is mainly restricted to head structures and demarcates neural tissues and derivatives. Strong expression is observed in the eyes (Fig. 2C-J, -K, -L; green arrow) and the prosencephalon (Fig. 2C-J, -K, -L, -M; red arrow).

Our results show, that in *Xenopus laevis rmnd5* transcripts and Rmnd5 protein are present continuously within the investigated time frame. The localisation of transcripts in neuronal ectoderm, prospective prosencephalon, the eyes and subpopulations of the skin suggests a function during neural development.

### Rmnd5 is important for proper early development of the embryonic forebrain

**Fig 3:**
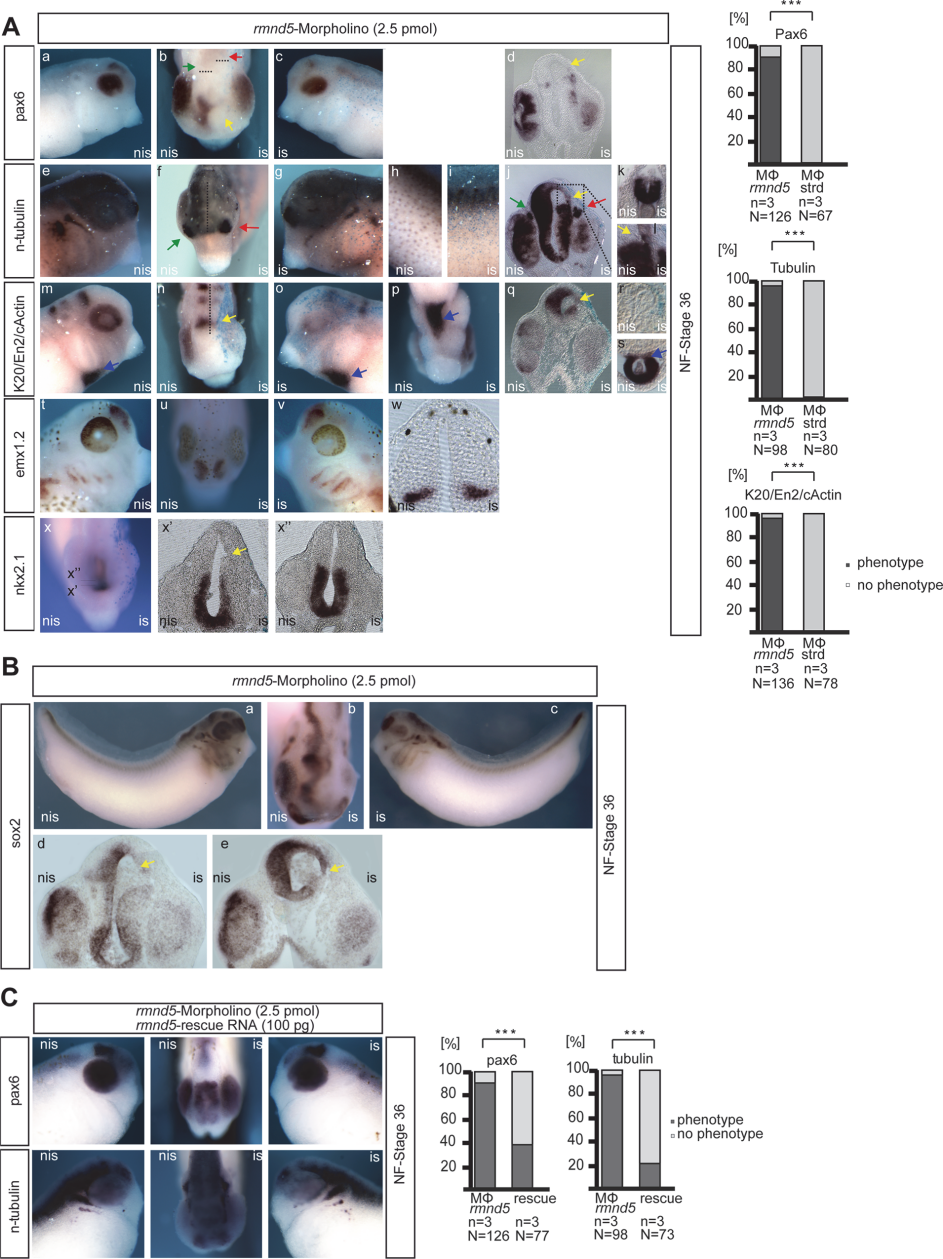
The CTLH complex functions during early embryonic neurogenesis. (A) *rmnd5*-mo injected embryos were used for *in situ* hybridisation with indicated marker probes; pax6 (upper lane), n-tubulin (middle lane), k20/en22/rx1/c-actin, emx1.2, nkx2.1 (bottom lanes). Abbreviations: IS, injected side; NIS, non-injected side. Quantitative representation of phenotypes are presented as a bar graph (percent embryos with phenotype to total amount (%); black, phenotype; grey, no phenotype); n = number of independent experiments, N = number of injected embryos analysed for respective marker, *P ≤0.05, ***≤0.001 (Chi Square test). (B) As (A) with *sox2* as probe. (C) Xenopus embryos co-injected with *rmnd5* morpholino (2.5 pmol/embryo) and synthetic capped RNA (100 pg/embryo) were used for *in situ* hybridisation and quantified as shown in A.

In order to analyze the function of Rmnd5, we inhibit the translation of *rmnd5* via injection of targeted antisense morpholino oligonucleotides. We injected 2.5 pmol *rmnd5*-morpholino together with 100 pg synthetic beta-*gal* RNA as a tracer into one cell of two-cell stage embryos and fixed them at NF stage 32–34. For further analysis, embryos were subjected to whole-mount *in situ* hybridisation (WMISH) after fixation and beta-*Gal* staining. A set of probes was employed to cover different states of differentiation and morphogenesis during the development of the central nervous system. Noticeable, loss of Rmnd5 function suppresses mostly the proper development of the mesencephalon, the prosencephalon and the eye. For example *pax6* expression at stage 34 is specific to amacrine and bipolar cells of the retina, epithelium of the lens, prospective prosencephalon and interneurons [22]. As a prominent phenotype, expression of *pax6* in the *rmnd5-*morpholino injected side (IS) is completely absent from the prospective prosencephalon (Fig. 3A-B, yellow arrow) while expression in the mid-/hindbrain was shifted to posterior (Fig. 3A-B, compare green/ red arrow). In a frontal section the lack of *pax6* signal in the dorsal area of the prospective prosencephalon is obvious, since the respective tissue is almost absent (Fig. 3A-D, yellow arrow). Thus, the lack of expression can be observed with all other marker genes (Fig. 3A-J, -L, [n-*tubulin*],-n,-q [*en2*],-x’ [*nkx2*.*1*] and Fig. 3B, -B, -D, -E, [*sox2*]; yellow arrow). Strikingly, the expression of n-*tubulin* in subpopulations of skin cells is also severely reduced (Fig. 3A-H, -I), while expression in the prosepective spinal cord is not altered (Fig. 3A-K). In contrast, structures arising from placodes such as the olfactory placodes (Fig. 3A-F, green/red arrow) and the trigeminal nerve (3A-J, green/red arrow) or of mesodermal origin such as the heart tube (3A-M, -O, -P, -S, c-Actin, blue arrow) appear normal. The non-injected sides (NIS) of these embryos and standard morpholino injected embryos (S2 Fig.) do not expose such phenotypes. To control for off-target effects of the *rmnd5* morpholino, we performed rescue experiments, injecting a synthetic *rmnd5* RNA that cannot be targeted by the *rmnd5*-morpholino. In the presence of the rescuing RNA, the *rmnd5* knockdown phenotype can be partially restored (Fig. 3C). Together our data suggests a role of Rmnd5 during the early development of dorsal areas of the pro- and mesencephalon, the eyes and most likely the ciliated cells of the skin. Its specific expression in these territories strongly supports a direct function of *rmnd5* during these processes (Fig. 2C-E,-H, -J, -K, -L, -M).

### Rmnd5 is part of an ubiquitin-ligase complex

**Fig 4:**
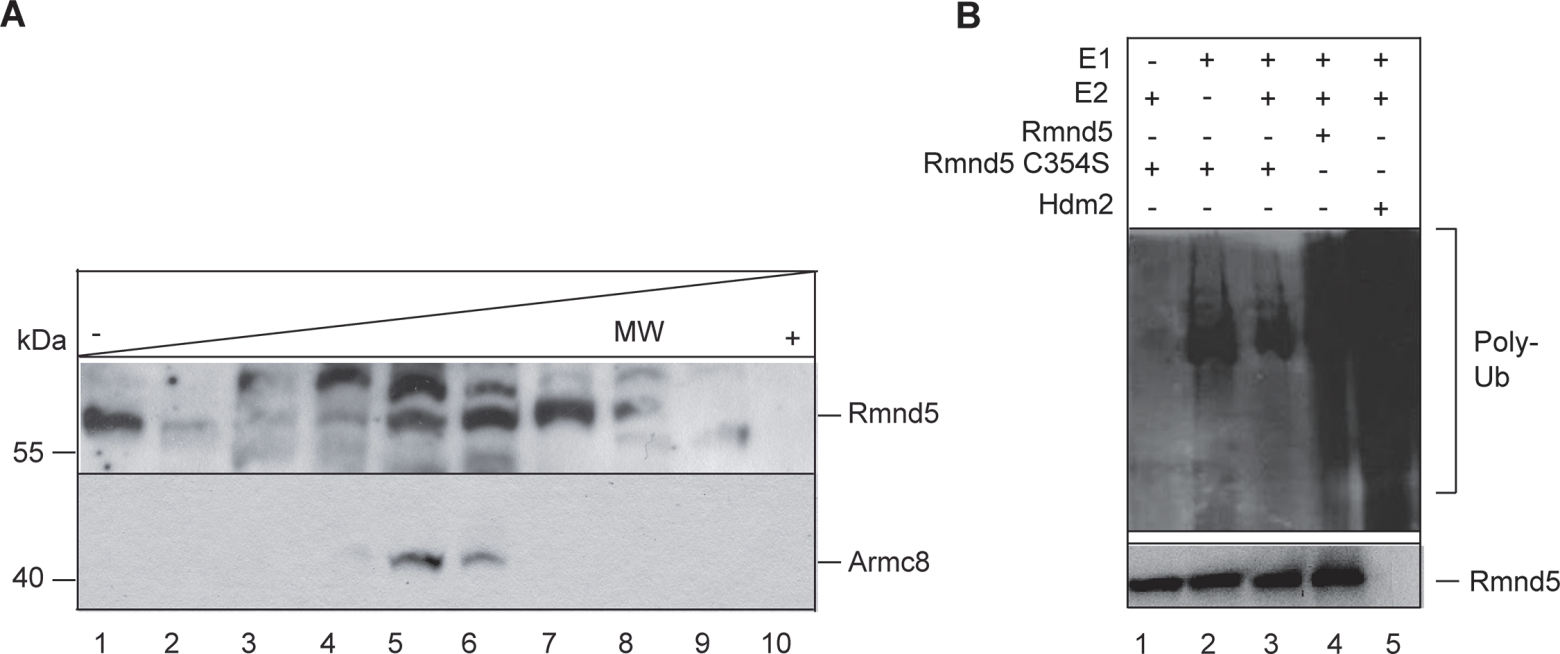
Rmnd5 is part of an ubiquitin ligase complex. (A) Glycerol step gradient of *Xenopus laevis* NF stage 36 embryo lysates. Molecular mass (MW) standard: albumin (67 kDa), fraction 1, 2; LDH (140 kDa), fraction 4; catalase (232 kDa), fraction 6,7. Western blot analysis with α-RMND5A (Rmnd5; upper panel) (1:1000) and α-ARMC8 (lower panel) (1:1000). (B) *In vitro* polyubiquitination assay with recombinant *Xenopus* Rmnd5 and Rmnd5-C354S (lane 3, 4). Reactions are performed in the presence (+) or absence (-) of E1 (lane 1), E2 (lane 2) and purified Rmnd5 protein. HDM2 is used as a positive control (lane 5). Polyubiquitination (Poly-Ub) is detected with α-HA and α-RMND5A as control.

In *Saccharomyces cerevisiae* we have shown that the Rmnd5 ortholog Gid2 is part of a specific ubiquitin-ligase complex targeting key gluconeogenic enzymes for polyubiquitination [3, 4]. In higher organisms the function of the CTLH-complex remains enigmatic. Several lines of evidence support the hypothesis that Rmnd5 functions as an ubiquitin-ligase as well. First, the non-canonical RING domains in Gid2/RMND5 and in Gid9/MAEA are evolutionary highly conserved. Second, human RMND5B interacts with several ubiquitin-conjugating enzymes (E2s) [16]. And finally, several protein interaction partners are putative CTLH complex substrates and are degraded by the 26S proteasome [23]. Therefore, we tested whether *Xenopus laevis* Rmnd5 is part of a high molecular mass protein complex and whether it exhibits ubiquitin-ligase activity. *Xenopus laevis* stage 36 embryo lysates were subjected to glycerol density centrifugation (Fig. 4A). Fractionation revealed that Rmnd5 is mostly present in fraction 6 and 7 at 200–300 kDa and fraction 1 at 50 kDa. The predicted molecular mass of Rmnd5 is 44 kDa; the protein appears to be partially associated with other proteins most probably in the context of the CTLH complex. Another subunit of the human CTLH-complex is ARMC8 [8]. The *Xenopus* ortholog has a predicted molecular mass of 40 kDa. However, it is detected in fraction 5 and 6 (150–250 kDa, lower panel) and thus partially cofractionates with Rmnd5 under these conditions. We conclude, that Rmnd5 cofractionates with known subunits of the CTLH complex and thus most likely is an integral part of it. In a next step, we wanted to test if Rmnd5 has E3 ubiquitin-ligase activity. Fig. 4B shows the result of an *in vitro* polyubiquitination assay [4]. Only in the presence of active *Xenopus laevis* Rmnd5 a strong polyubiquitination signal was detected. The Rmnd5 mutant protein containing a mutation in the conserved RING cysteine residue (C354S) and the respective negative controls do not show any autoubiquitination (Fig. 4B, lane 1, 2, 3). As a positive control, the human ubiquitin ligase HDM2 was included in the test. We conclude that vertebrate Rmnd5 possesses E3 ligase activity and is part of a multimeric protein complex, most probably the CTLH complex. Given the close evolutionary relationship between the Gid2/Rmnd5 proteins we asked whether *Xenopus laevis* Rmnd5 is able to complement a *gid2Δ* deletion phenotype in *Saccharomyces cerevisiae*. We expressed a yeast codon optimized *Xenopus laevis* rmnd5 (sequence available upon request) in a *gid2Δ* deletion strain and measured the half-life of the gluconeogenic enzyme FBPase. Even elevated amounts of Rmnd5 are not able to rescue the *Δgid2* phenotype (S1 Fig.). Additionally, no interaction between Rmnd5 and the yeast Gid complex can be measured. Despite the high degree of conservation, the structural differences of the protein binding domains appear to be too strong to allow incorporation of Rmnd5 into the yeast complex.

Taken together, we show for the first time that Rmnd5 of a higher organism is part of an ubiquitin-ligase complex. Unexpectedly, the suppression of *rmnd5* function during embryonic development leads to malformations specifically in dorsal parts of the pros- and mesencephalon, the eyes and of ciliated cells of the skin. Therefore, the accumulation of an unknown Rmnd5 substrate might disturb the proper regulation of e.g. neural progenitor proliferation, cell specification, neuronal differentiation, cell migration or ciliogenesis. Recently, the human transcription factor Sox2 was shown to interact with the entire CTLH-complex [24]. Sox2 is essential for the maintenance of neural stem cell pluripotency with a key role during the development of pituitary, forebrain and the eyes [25, 26] and shows a similar expression pattern as *rmnd5* (compare Fig. 2C and Fig. 3B) We therefore assume that the CTLH complex regulates Sox2 stability and thus may explain the manifestation of Rmnd5 dependent malformations of neural structures in our animal model and the patient [18].

## Material and Methods

### Capped mRNA and morpholino injections

Capped *rmnd5* m-RNA was generated using the mMESSAGE mMACHINE kit (Ambion, Austin, TX). Not1 linearized pTP251 was used as a template for SP6 transcription and 5 nl of capped mRNA (~2.5 ng) was injected into one blastomere of a 2-cell stage embryo together with rmnd5 morpholino. 25-mer morpholinos (MOs; Gene Tools, LLC Philomath, Oregon) were designed to target the ATG translation start site for *rmnd5* (NM_001092807.1) mRNA transcripts (sequence: GCTCCACCGTGCCGCACTGATCCAT). A mismatch standard MO was used as control (sequence: CCTCTTACCTCAGTTACAATTTATA). Both were injected (2.5 pmol) into one blastomere of 2-cell stage embryos.

### Glycerol Density Gradient Fractionation

100 μl aliquots of embryo extract were layered on top of a glycerol step gradient in lysis buffer (950 μl each of 50%, 40%, 30%, 20 and 10% of glycerol) and centrifuged at 340000 g in a SW60TI rotor (Beckman Coulter) for 22h at 16°C. Thereafter, 330 μl fractions were collected, total protein precipitated with trichloroacetic acid (10% final), solubilised in urea buffer and analyzed by Western blotting.

### Purification of recombinant His-tagged protein

Expression and purification of recombinant Rmnd5 was performed as described before [27].

### RNA preparation and reverse transcription

RNA was prepared from whole *Xenopus* embryos using a Qiagen RNeasy Kit following the instructions provided by the manufacturers. First strand cDNA was prepared from 500 ng total RNA using oligo-dT or random primer and reverse transcriptase (Gibco).

### Whole mount *in situ* hybridization and RT-PCR

To analyze the spatio-temporal expression of *rmnd5* during *Xenopus laevis* embryogenesis, a DIG-labeled *antisense* RNA probe was generated by linearizing pTP221 with HindIII-HF (NEB) and *in vitro* transcription with T7 RNA Polymerase (Roche) as described before [28]. *Xenopus* embryos were fixed at consecutive developmental stages and whole-mount *in situ* hybridization was carried out as previously described [29]. Embryos probed with antisense RNAs of n-*tubulin*, *pax6*, and *rx1*-*en2*-*krox20*, *sox2*, *emx1*.*2*, *nkx2* and *rmnd5* respectively were vibratome sectioned (30 μm) and photographed.

### Oligonucleotides and plasmids

Plasmids and primers used in this work are listed in Table 1 and Table 2, respectively and are available upon request. Plasmid pTP250 contains the *Xenopus laevis rmnd5* ORF amplified from pTP221 with primers TP250fwd and TP250rev, inserted via XhoI and HindIII into pEGFP-C1 (Clontech). pTP221 contains the *Xenopus laevis rmnd5* ORF amplified from cDNA, which was inserted into EcoRI/XhoI linearized pTP213 by yeast homologous recombination and selection on CSD—URA medium. pTP230 is a pCA528 derivative containing *Xenopus laevis rmnd5* that was cut from pTP221 and inserted into pCA528via XhoI and BamHI. pTP241 is a pTP230 derivative with a C354S mutation in the RMND5A gene SOMA-generated with primer TP226 [21]. Site-directed mutagenesis of plasmids was performed using SOMA [21].

**Table 1:** Primers used in this study.

Primer	Description	Reference
TP251	CCCATCGATTCGAATTCAATGGACCAATGTGGTACCGTAGAACGGGAGCTGGAGAAGGTGC	This work
TP250fwd	CATCATCTCGAGAAGATCAGTGCGGCACGGTG	This work
TP250rev	CATCATAAGCTTGAGTCAGAAAAAGATTTGTTTGGCGTCTC	This work
TP221fwd	TTGCAGGATCCCATCGATTCGAATTCAATGGATCAGTGCGGCACGGTG	This work
TP221rev	TACGACTCACTATAGTTCTAGAGGCTCGAGTCAGAAAAAGATTTGTTTGGCG	This work
TP226	CCTATGAAGCTTGTTTCCGGACACATTATATCC	This work
XL-Rmd5fwd	GAGCGGGAGCTGGAGAAGGT	This work
XL-Rmd5rev	CAGCCATCAATCCCCACGCT	This work
XL-ODC-F	GCCATTGTGAAGACTCTCTCCATTC	This work
XL-ODC-R	TTCGGGTGATTCCTTGCCAC	This work

**Table 2:** Plasmids used in this study.

Name	Description	Source
pTP230	pCA528 based vector with the *Xenopus laevis* open reading frame *rmnd5* (NM_001092807.1)	This work
pTP241	pTP230 with a C354-S mutation in *rmnd5*	This work
pTP221	pTP213 containing *Xenopus laevis rmnd5* (NM_001092807.1)	This work
pTP213	pCS2^+^ containing CEN/ARS and URA3 gene from *S*. *cerevisiae*	[21]
pCA528	E.coli expression vector for T7-promoted expression of His_6_-SUMO fusion proteins	[30]
pTP250	pEGFP-C1 containing *rmnd5* (Xenopus laevis)	This work
pTP224	pEGFP-C1 containing RMND5b (homo sapiens)	[21]
pTP225	pEGFP-C1 containing RMND5a (homo sapiens)	[21]
pTP251	*Rmnd5* binding site silently mutated in pTP221	This work

### Organisms and maintenance

Frogs were obtained from a commercial supplier (NASCO, USA). Production and rearing of embryos was performed as described previously [29] and embryos were maintained at 15°C and staged according to Nieuwkoop and Faber. All procedures were performed according to guidelines set by the german animal use and care laws (Tierschutzgesetz) and approved by the german state administration Saxony-Anhalt (Projekt/AZ: 42502-3-600 MLU). HEK293 cells were maintained in Dulbecco’s modified Eagle’s medium supplemented with 10% fetal calf serum (v/v). Transfections were performed using Lipofectamin 2000 (Invitrogen). For microscopy studies cells were grown on cover slips pretreated with poly-l-lysine solution, 0,1% (Sigma-Aldrich) for 5 min.

### Western blotting

Western blotting was performed as described earlier [4]. Embryo extracts were prepared by homogenizing embryos with an injection needle (Braun, 0,7 × 30 mm) in one pellet volume of lysis buffer (20 mM Tris (pH8), 100 mM NaCl, 1mM EDTA, 0,5% Triton X-100, 10% glycerol, 0,1 mM PMSF, 10 μg/ml leupeptin, 1 μg/ml pepstatinA, 5 μg/ml aprotinin). Total protein was determined with the Bradford assay to load 20–30 μg total protein per lane. Antibodies were purchased from Abcam (Polyclonal ARMC8; WB 1:1000), Novus Biological (Polyclonal RMND5A; WB 1:1000).

### Autoubiquitination assay

A 25 μl ubiquitination reaction contained 0.25 μg of E1 (yeast), 0.6 μg of UbcH5b, 10 μg of HA-ubiquitin, 1 μl of energy regeneration solution (all BostonBiochem, Cambridge), 2 μl ATP (100 mM, pH7.5), 2.5 μl ubiquitin reaction buffer (500 mM Tris-HCl, pH7.5, 500 mM NaCl, 100 mM MgCl2, 10 mM DTT, and 250 μM ZnCl2) and 2.25 μg of purified Rmnd5 or HDM2 (Enzo Lifescience) as a positive control. The reactions were incubated at 30°C for 3 h. Ubiquitination of protein was monitored by Western blot analysis with polyclonal anti-HA antibody (Sigma).

## Supporting Information

S1 Fig Complementation assay of *Xenopus laevis* Rmnd5 in yeast *Δgid2*. Catabolite degradation of fructose-1,6-bisphosphatase (FBPase) in yeast. *Δgid2* (YWO0906) was transformed with plasmid pRM41 harbouring V5-tagged *rmnd5*. YWO0906 and YWO2023 (containing *GID2-V5*) were transformed with empty plasmid pRS426 as controls. Cells were grown for 12h in synthetic complete medium without uracil containing 2% glucose. After addition of 2% glucose 1.5 OD_600_ of cells were taken at the indicated time points. Total protein was extracted and precipitated with trichloroacetic acid, resuspended in urea buffer and subjected to Western blot analysis with polyclonal FBPase antiserum, Pgk antibody (Molecular probes) and V5 antibody (Thermo Scientific), respectively. Digital data were quantified using TotalLab Quant and Excel; FBPase signals were normalised with 3-phosphoglycerate kinase (Pgk) (A) Representative Western blot of a complementation experiment. (B) Quantification of FBPase signal after glucose addition. Graphs include data from n = 10 (Rmnd5, blue), n = 7 (gid2 = *Δgid2*, red) and n = 5 (GID2, purple) experiments, respectively.
(TIF)

S2 Fig The CTLH complex functions during early embryonic neurogenesis. As Fig. 3 with standard morpholino injected embryos
(TIF)

## References

[1] Regelmann J, Schule T, Josupeit FS, Horak J, Rose M, Entian KD, et al Catabolite degradation of fructose-1,6-bisphosphatase in the yeast Saccharomyces cerevisiae: a genome-wide screen identifies eight novel GID genes and indicates the existence of two degradation pathways. Mol Biol Cell. 2003;14(4):1652–63. Epub 2003/04/11. 10.1091/mbc.E02-08-0456 PubMed PMID: . 12686616 PMC153129

[2] Menssen R, Schweiggert J, Schreiner J, Kusevic D, Reuther J, Braun B, et al Exploring the Topology of the Gid Complex, the E3 Ubiquitin Ligase Involved in Catabolite-induced Degradation of Gluconeogenic Enzymes. J Biol Chem. 2012;287(30):25602–14. Epub 2012/05/31. 10.1074/jbc.M112.363762 PubMed PMID: 22645139 PMC3408164

[3] Braun B, Pfirrmann T, Menssen R, Hofmann K, Scheel H, Wolf DH. Gid9, a second RING finger protein contributes to the ubiquitin ligase activity of the Gid complex required for catabolite degradation. FEBS Lett. 2011. Epub 2011/11/03. S0014-5793(11)00789-7 [pii] 10.1016/j.febslet.2011.10.038 PubMed PMID: . 22044534

[4] Santt O, Pfirrmann T, Braun B, Juretschke J, Kimmig P, Scheel H, et al The yeast GID complex, a novel ubiquitin ligase (E3) involved in the regulation of carbohydrate metabolism. Mol Biol Cell. 2008;19(8):3323–33. Epub 2008/05/30. E08-03-0328 [pii] 10.1091/mbc.E08-03-0328 PubMed PMID: . 18508925 PMC2488282

[5] Sommer T, Wolf DH. The ubiquitin-proteasome-system. Biochim Biophys Acta. 2014;1843(1):1 Epub 2013/09/24. 10.1016/j.bbamcr.2013.09.009 PubMed PMID: . 24055503

[6] Deshaies RJ, Joazeiro CA. RING domain E3 ubiquitin ligases. Annu Rev Biochem. 2009;78:399–434. Epub 2009/06/06. 10.1146/annurev.biochem.78.101807.093809 PubMed PMID: . 19489725

[7] Francis O, Han F, Adams JC. Molecular Phylogeny of a RING E3 Ubiquitin Ligase, Conserved in Eukaryotic Cells and Dominated by Homologous Components, the Muskelin/RanBPM/CTLH Complex. PloS one. 2013;8(10):e75217 Epub 2013/10/22. 10.1371/journal.pone.0075217 PubMed PMID: 24143168 PMC3797097

[8] Kobayashi N, Yang J, Ueda A, Suzuki T, Tomaru K, Takeno M, et al RanBPM, Muskelin, p48EMLP, p44CTLH, and the armadillo-repeat proteins ARMC8alpha and ARMC8beta are components of the CTLH complex. Gene. 2007;396(2):236–47. Epub 2007/05/01. S0378-1119(07)00120-5 [pii] 10.1016/j.gene.2007.02.032 PubMed PMID: . 17467196

[9] Umeda M, Nishitani H, Nishimoto T. A novel nuclear protein, Twa1, and Muskelin comprise a complex with RanBPM. Gene. 2003;303:47–54. Epub 2003/02/01. S0378111902011538 [pii]. PubMed PMID: . 12559565 10.1016/s0378-1119(02)01153-8

[10] Tomastikova E, Cenklova V, Kohoutova L, Petrovska B, Vachova L, Halada P, et al Interactions of an Arabidopsis RanBPM homologue with LisH-CTLH domain proteins revealed high conservation of CTLH complexes in eukaryotes. BMC plant biology. 2012;12:83 Epub 2012/06/09. 10.1186/1471-2229-12-83 PubMed PMID: . 22676313 PMC3464593

[11] Gerlitz G, Darhin E, Giorgio G, Franco B, Reiner O. Novel functional features of the Lis-H domain: role in protein dimerization, half-life and cellular localization. Cell Cycle. 2005;4(11):1632–40. Epub 2005/11/01. PubMed PMID: . 16258276 10.4161/cc.4.11.2151

[12] Emes RD, Ponting CP. A new sequence motif linking lissencephaly, Treacher Collins and oral-facial-digital type 1 syndromes, microtubule dynamics and cell migration. Human molecular genetics. 2001;10(24):2813–20. Epub 2001/12/06. PubMed PMID: . 11734546 10.1093/hmg/10.24.2813

[13] Tagnaouti N, Loebrich S, Heisler F, Pechmann Y, Fehr S, De Arcangelis A, et al Neuronal expression of muskelin in the rodent central nervous system. BMC neuroscience. 2007;8:28 Epub 2007/05/04. 10.1186/1471-2202-8-28 PubMed PMID: 17474996 PMC1876237

[14] Heisler FF, Loebrich S, Pechmann Y, Maier N, Zivkovic AR, Tokito M, et al Muskelin regulates actin filament- and microtubule-based GABA(A) receptor transport in neurons. Neuron. 2011;70(1):66–81. Epub 2011/04/13. 10.1016/j.neuron.2011.03.008 PubMed PMID: 21482357 PMC3101366

[15] Tomaru K, Ueda A, Suzuki T, Kobayashi N, Yang J, Yamamoto M, et al Armadillo Repeat Containing 8alpha Binds to HRS and Promotes HRS Interaction with Ubiquitinated Proteins. Open Biochem J. 2010;4:1–8. Epub 2010/03/13. 10.2174/1874091X01004010001 PubMed PMID: 20224683 PMC2835868

[16] van Wijk SJ, de Vries SJ, Kemmeren P, Huang A, Boelens R, Bonvin AM, et al A comprehensive framework of E2-RING E3 interactions of the human ubiquitin-proteasome system. Mol Syst Biol. 2009;5:295 Epub 2009/08/20. msb200955 [pii] 10.1038/msb.2009.55 PubMed PMID: . 19690564 PMC2736652

[17] Li J, Chen Y, Qin X, Wen J, Ding H, Xia W, et al MiR-138 downregulates miRNA processing in HeLa cells by targeting RMND5A and decreasing Exportin-5 stability. Nucleic acids research. 2014;42(1):458–74. Epub 2013/09/24. 10.1093/nar/gkt839 PubMed PMID: 24057215 PMC3874158

[18] Vogel TW, Manjila S, Cohen AR. Novel neurodevelopmental disorder in the case of a giant occipitoparietal meningoencephalocele. Journal of neurosurgery Pediatrics. 2012;10(1):25–9. Epub 2012/06/12. 10.3171/2012.3.PEDS11559 PubMed PMID: . 22681319

[19] Notredame C, Higgins DG, Heringa J. T-Coffee: A novel method for fast and accurate multiple sequence alignment. Journal of molecular biology. 2000;302(1):205–17. Epub 2000/08/31. 10.1006/jmbi.2000.4042 PubMed PMID: . 10964570

[20] Dereeper A, Guignon V, Blanc G, Audic S, Buffet S, Chevenet F, et al Phylogeny.fr: robust phylogenetic analysis for the non-specialist. Nucleic acids research. 2008;36(Web Server issue):W465–9. Epub 2008/04/22. 10.1093/nar/gkn180 PubMed PMID: 18424797 PMC2447785

[21] Pfirrmann T, Lokapally A, Andreasson C, Ljungdahl P, Hollemann T. SOMA: a single oligonucleotide mutagenesis and cloning approach. PloS one. 2013;8(6):e64870 Epub 2013/06/12. 10.1371/journal.pone.0064870 PubMed PMID: 23750217 PMC3672168

[22] Hirsch N, Harris WA. Xenopus Pax-6 and retinal development. Journal of neurobiology. 1997;32(1):45–61. Epub 1997/01/01. PubMed PMID: . 8989662

[23] Suzuki T, Ueda A, Kobayashi N, Yang J, Tomaru K, Yamamoto M, et al Proteasome-dependent degradation of alpha-catenin is regulated by interaction with ARMc8alpha. Biochem J. 2008;411(3):581–91. Epub 2008/01/25. 10.1042/BJ20071312 PubMed PMID: . 18215130

[24] Cox JL, Wilder PJ, Gilmore JM, Wuebben EL, Washburn MP, Rizzino A. The SOX2-interactome in brain cancer cells identifies the requirement of MSI2 and USP9X for the growth of brain tumor cells. PloS one. 2013;8(5):e62857 Epub 2013/05/15. 10.1371/journal.pone.0062857 PubMed PMID: 23667531 PMC3647065

[25] Kelberman D, de Castro SC, Huang S, Crolla JA, Palmer R, Gregory JW, et al SOX2 plays a critical role in the pituitary, forebrain, and eye during human embryonic development. The Journal of clinical endocrinology and metabolism. 2008;93(5):1865–73. Epub 2008/02/21. 10.1210/jc.2007-2337 PubMed PMID: 18285410 PMC3479085

[26] Rizzino A. Sox2 and Oct-3/4: a versatile pair of master regulators that orchestrate the self-renewal and pluripotency of embryonic stem cells. Wiley interdisciplinary reviews Systems biology and medicine. 2009;1(2):228–36. Epub 2009/12/18. 10.1002/wsbm.12 PubMed PMID: 20016762 PMC2794141

[27] Panavas T, Sanders C, Butt TR. SUMO fusion technology for enhanced protein production in prokaryotic and eukaryotic expression systems. Methods Mol Biol. 2009;497:303–17. Epub 2008/12/25. 10.1007/978-1-59745-566-4_20 PubMed PMID: . 19107426

[28] Cornesse Y, Pieler T, Hollemann T. Olfactory and lens placode formation is controlled by the hedgehog-interacting protein (Xhip) in Xenopus. Developmental biology. 2005;277(2):296–315. Epub 2004/12/25. 10.1016/j.ydbio.2004.09.016 PubMed PMID: . 15617676

[29] Hollemann T, Bellefroid E, Pieler T. The Xenopus homologue of the Drosophila gene tailless has a function in early eye development. Development. 1998;125(13):2425–32. Epub 1998/06/04. PubMed PMID: . 9609825 10.1242/dev.125.13.2425

[30] Andreasson C, Fiaux J, Rampelt H, Mayer MP, Bukau B. Hsp110 is a nucleotide-activated exchange factor for Hsp70. J Biol Chem. 2008;283(14):8877–84. Epub 2008/01/26. 10.1074/jbc.M710063200 PubMed PMID: . 18218635

