# Supplementary figures and images for: RMND5 from Xenopus laevis Is an E3 Ubiquitin-Ligase and Functions in Early Embryonic Forebrain Development
Source: PLoS One. 2015 Mar 20;10(3):e0120342. doi: 10.1371/journal.pone.0120342 (PMC4368662; doi:10.1371/journal.pone.0120342)

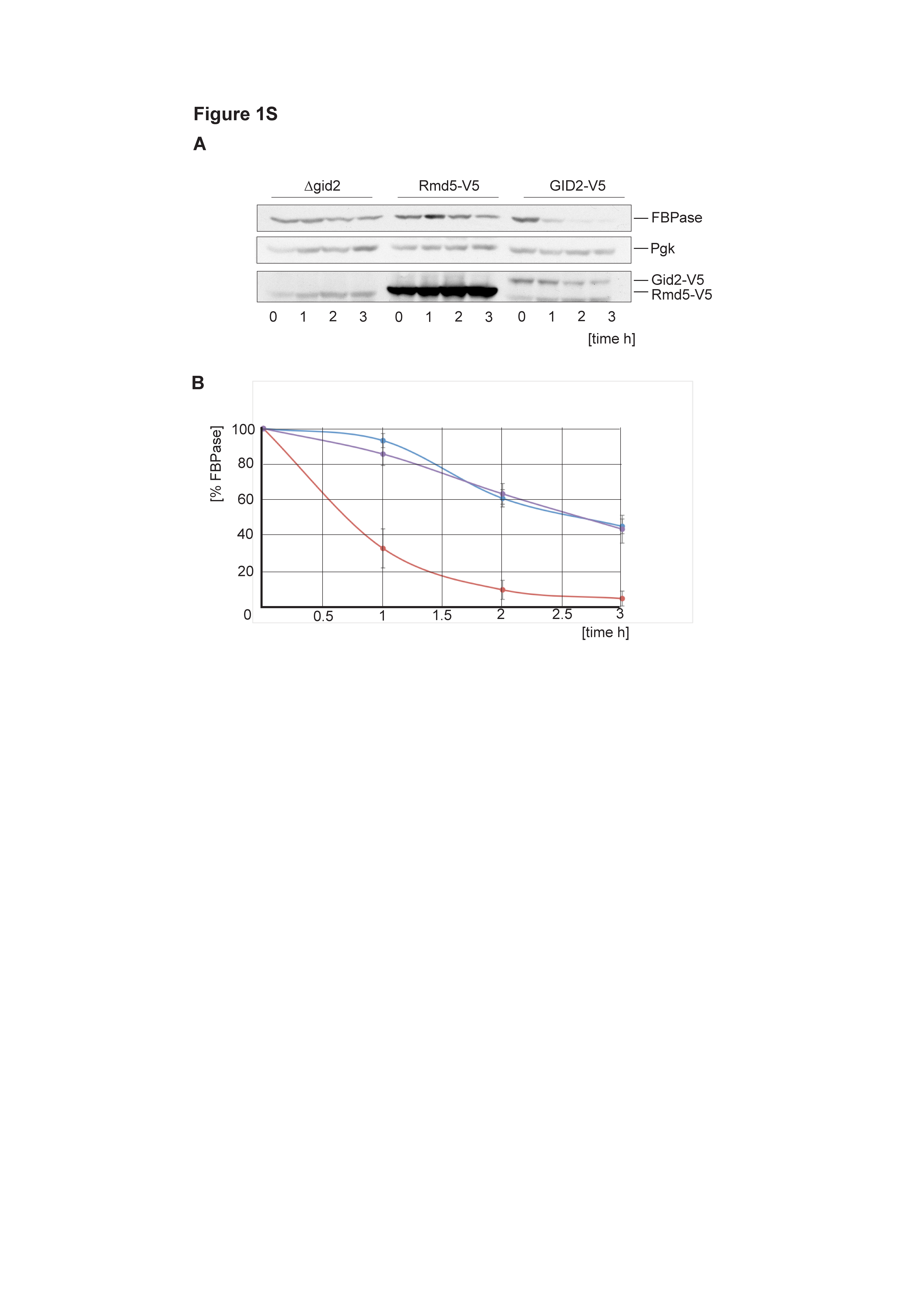

Supplement: S1 Fig — Catabolite degradation of fructose-1,6-bisphosphatase (FBPase) in yeast. Δgid2 (YWO0906) was transformed with plasmid pRM41 harbouring V5-tagged rmnd5. YWO0906 and YWO2023 (containing GID2-V5) were transformed with empty plasmid pRS426 as controls. Cells were grown for 12h in synthetic complete medium without uracil containing 2% glucose. After addition of 2% glucose 1.5 OD600 of cells were taken at the indicated time points. Total protein was extracted and precipitated with trichloroacetic acid, resuspended in urea buffer and subjected to Western blot analysis with polyclonal FBPase antiserum, Pgk antibody (Molecular probes) and V5 antibody (Thermo Scientific), respectively. Digital data were quantified using TotalLab Quant and Excel; FBPase signals were normalised with 3-phosphoglycerate kinase (Pgk) (A) Representative Western blot of a complementation experiment. (B) Quantification of FBPase signal after glucose addition. Graphs include data from n = 10 (Rmnd5, blue), n = 7 (gid2 = Δgid2, red) and n = 5 (GID2, purple) experiments, respectively. (TIF) [file pone.0120342.s001.tif]

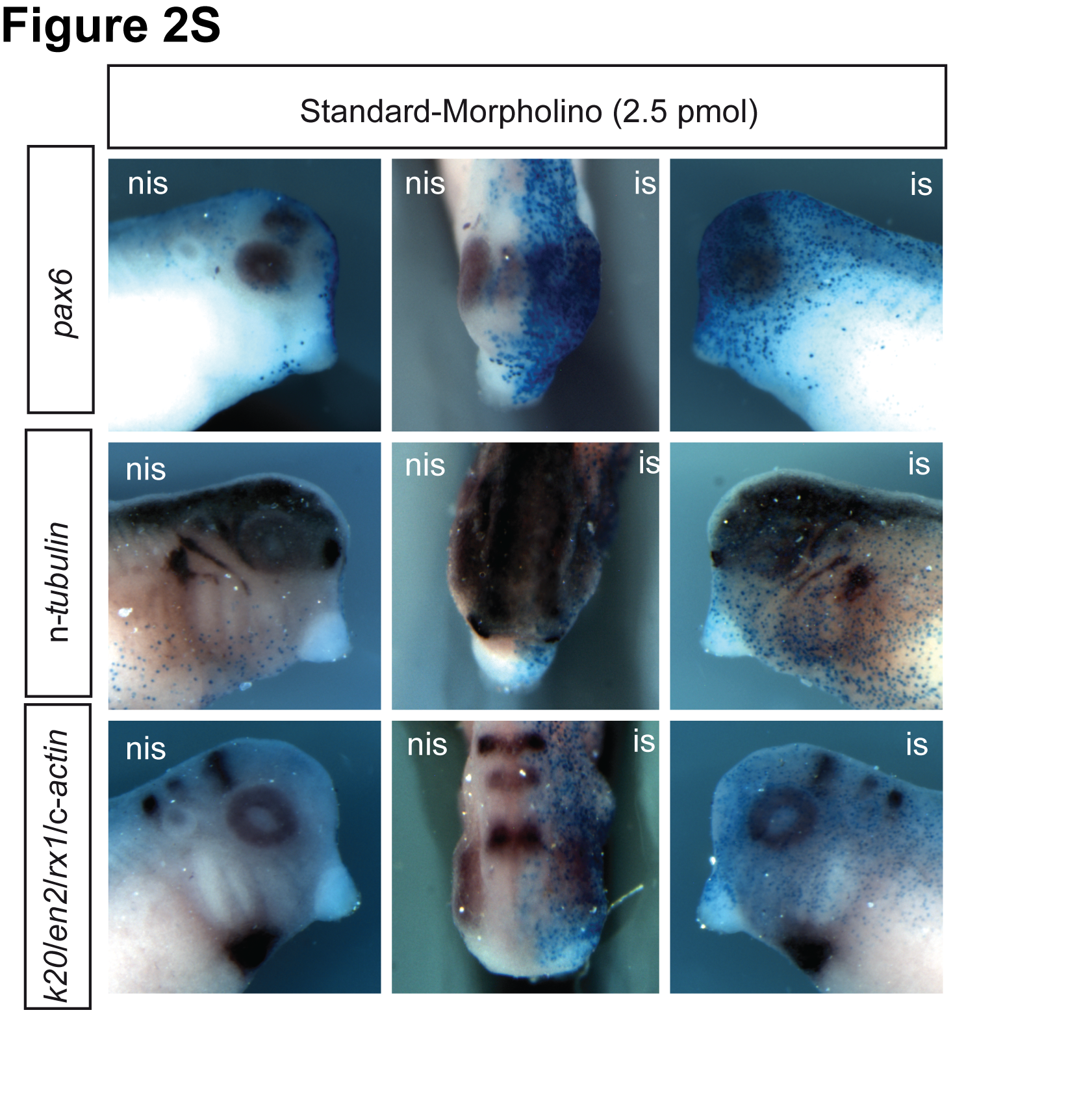

Supplement: S2 Fig — As Fig. 3 with standard morpholino injected embryos (TIF) [file pone.0120342.s002.tif]
